# Sarcoidosis with bone involvement mimicking metastatic disease at ^18^F-FDG PET/CT: problem solving by diffusion whole-body MRI

**DOI:** 10.3332/ecancer.2015.537

**Published:** 2015-05-07

**Authors:** Giorgio Conte, Fabio Zugni, Marco Colleoni, Giuseppe Renne, Massimo Bellomi, Giuseppe Petralia

**Affiliations:** 1 Department of Health Sciences, University of Milan, Via Antonio Di Rudini 8, Milan 20142, Italy; 2 Division of Medical Senology, European Institute of Oncology, Via Ripamonti 435, Milan 20141, Italy; 3 Division of Pathology, European Institute of Oncology, Via Ripamonti 435, Milan 20141, Italy; 4 Division of Radiology, European Institute of Oncology, Via Ripamonti 435, Milan 20141, Italy

**Keywords:** bone, metastasis, sarcoidosis, whole body imaging, diffusion weighted imaging

## Abstract

Bone involvement has been reported in 1–13% of patients with sarcoidosis. Both 18F-fluorodeoxyglucose (^18^F-FDG) Positron Emission Tomography/Computed Tomography (PET/CT) and conventional magnetic resonance imaging (MRI) are sensitive in detecting sarcoidosis bone lesions, but are not always reliable in differentiating sarcoidosis bone lesions from metastatic disease, thus often requiring bone biopsy. We describe the use of diffusion whole-body MRI for bone assessment in a patient with breast cancer and sarcoidosis, presenting with bone marrow lesions mimicking metastatic disease at ^18^F-FDG PET/CT. In our case, diffusion whole-body MRI represented a useful tool for bone assessment and overcame the limitation of ^18^F-FDG PET/CT in discriminating inflammatory bone marrow involvement from metastatic disease.

## Background

Sarcoidosis is a multisystem granulomatous disease of uncertain etiology, typically involving the lungs, lymph nodes, and central nervous system with bone involvement being reported in 1–13% (average 5%) of patients [1]. However, this percentage is derived from the detection of sarcoidosis bone lesions on radiographs as there is still a lack of large scale studies based on newer imaging techniques, such as MRI and ^18^F-FDG PET/CT that could investigate the incidence of sarcoidosis bone lesions in the whole skeleton, supporting the hypothesis that bone involvement may be more frequent in this disease.

^18^F-FDG PET/CT is highly sensitive in detecting granulomatous bone marrow infiltration, but an increased ^18^F-FDG uptake can mimic metastatic disease, reducing the specificity of ^18^FDG PET/CT when both sarcoidosis and a tumour which may develop bone metastases occur in the same patient [2]. Bone assessment in sarcoidosis patients is also performed using MRI, commonly relying on T1-weighted and T2-weighted images [3]. However, routine MRI is not reliable in differentiating sarcoidosis bone lesions from metastatic disease [4].

We describe the use of diffusion whole-body MRI for bone assessment in a patient with breast cancer and sarcoidosis, presenting with bone marrow lesions mimicking metastatic disease at ^18^F-FDG PET/CT. This case suggests that diffusion whole-body MRI may represent a helpful tool for bone assessment in cancer patients, without contrast administration and radiation dose, providing anatomical and functional information which may overcome the limitation of ^18^F-FDG PET/CT in discriminating inflammatory bone marrow involvement from metastatic disease.

## Case report

**Figure 1.**
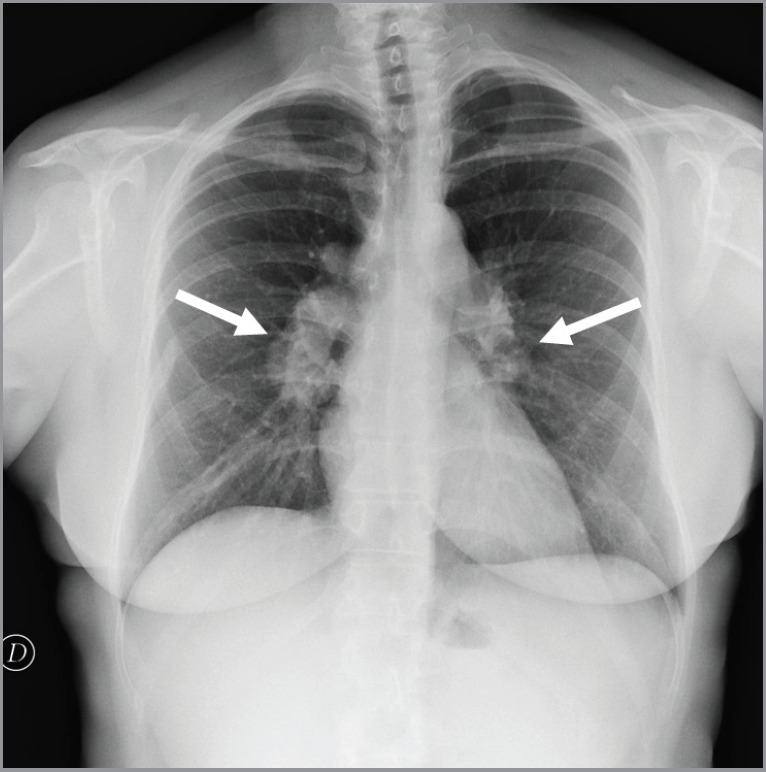
Chest x-ray showed bilateral hilar lymphadenopathies (arrows) suggestive for chronic granulomatous disease.

In October 2012, a 50-year-old woman presented with breast cancer. A chest x-ray and an abdominal ultrasound were performed for staging. The ultrasound was negative while the chest x-ray detected bilateral symmetric hilar lymphadenopathies, suggestive for granulomatous chronic disease, without suspicious lung lesions (Figure 1). The tumour was clinically staged as cT2N1. A left mastectomy with axillary lymph node dissection was performed. The pathology report showed ductal invasive carcinoma of 5.3 centimeters (pT3), with two out of 40 axillary lymph nodes involved (pN1), absence of oestrogen receptor (ER 0%) and progesterone receptors (PGR 0%), Ki-67 33%, and HER-2/neu expression at 95%.

**Figure 2.**
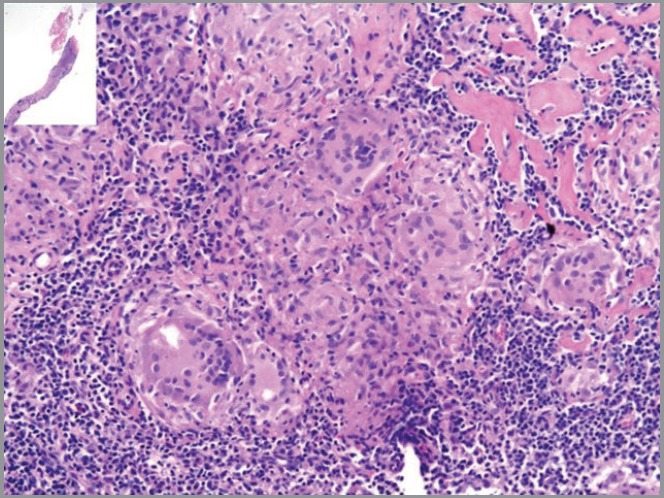
Histology of the groin lymph node. Photomicrograph of needle core biopsy specimen shows well-formed non-caseating confluent granulomas, and hyalinising focal fibrosis (H & E stain – x40). Special stains for organisms were negative.

In November 2012, whole-body ^18^F-FDG PET/CT for staging showed increased ^18^F-FDG-uptake in multiple supra-diaphragmatic and pelvic lymph nodes suggestive for chronic inflammatory granulomatous disease. Core-biopsy of a groin lymph node demonstrated non-necrotising granulomatous lymphadenitis suspicious for sarcoidosis (Figure 2). Special stains and culture for bacteria, mycobacteria, and fungus tested negative. Additionally, antineutrophil cytoplasmic antibody (ANCA) serologies, fungal serologies, and QuantiFERON tested negative.

In December 2012, after a rheumatological evaluation, stage I sarcoidosis was diagnosed and the patient was initiated on prednisone therapy (5 mg per day). Epirubicin and cyclophosphamide were administered as adjuvant chemotherapy for four cycles starting from December 2012, followed by Herceptin and Taxol for 12 weeks.

In March 2013, a total-body CT confirmed the mediastinal lymphadenopathies. It did not show the pelvic lymphadenopathies but demonstrated pulmonary micronodules in a perilymphatic distribution, which were considered related to sarcoidosis. The patient’s prednisone dose was increased to 7 mg per day.

In September 2013, a ^18^F-FDG PET/CT examination showed no significant variation in ^18^F-FDG uptake.

**Figure 3.**
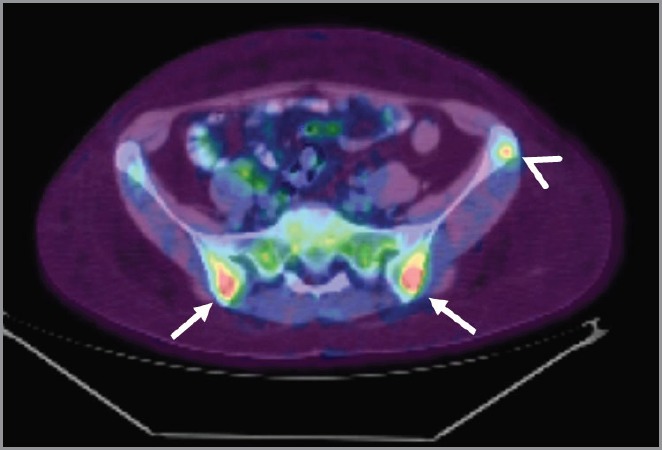
Pelvic bone lesions on ^18^F-FDG-PET/CT. ^18^F-FDG-PET/CT showed ill-defined lesions in the iliac bones (arrow) and a round-shaped lesion in the left iliac crest (arrowhead) with increased ^18^F-FDG-uptake.

**Figure 4.**
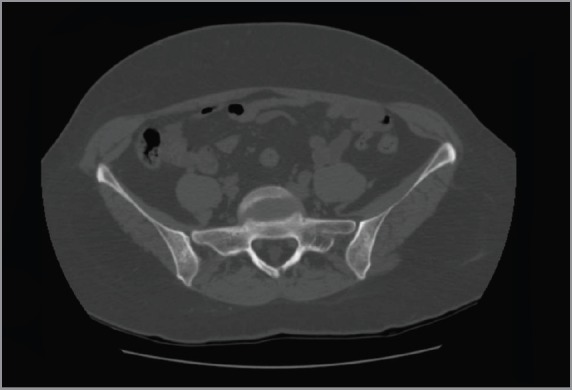
CT of the pelvis. Preliminary CT scan before CT-guided biopsy showed no evidence of abnormalities or where to target the biopsy.

In March 2014, the patient reported pain in sacroiliac region. No significant pathological signs were revealed at clinical examination. Laboratory markers used for the follow-up of sarcoidosis and breast cancer were normal (erythrocyte sedimentation rate, renal function, serum calcium, CEA 15.3). ^18^F-FDG PET/CT showed no significant variation in the mediastinum and the lungs but demonstrated the appearance of a diffuse bone marrow ^18^F-FDG uptake in the iliac bones (Figure 3), which was reported as suggestive for both metastatic disease or sarcoidosis bone involvement. The patient was therefore referred to CT-guided biopsy, but preliminary CT scan did not show any suspicious bone lesion to biopsy (Figure 4). The radiologist decided to avoid blind biopsy and referred the patient to diffusion whole-body MRI.

In August 2014, diffusion whole-body MRI was performed at 1.5-T system (Avanto, Siemens Healthcare) equipped with multiple surface coils and capable of continuous table movement imaging. The MR protocol consisted of axial diffusion-weighted images (b-values, 50–900 s/mm^2^) covering from head to knee, and additional anatomical images including: sagittal T1-weighted turbo spin-echo and T2-weighted short-tau inversion recovery (STIR) on the spine, axial T1-weighted gradient-echo and T2-weighted STIR images covering from head to knee. No contrast agent was administered. The time of acquisition for the whole-body diffusion MRI was of about 50 minutes.

**Figure 5.**
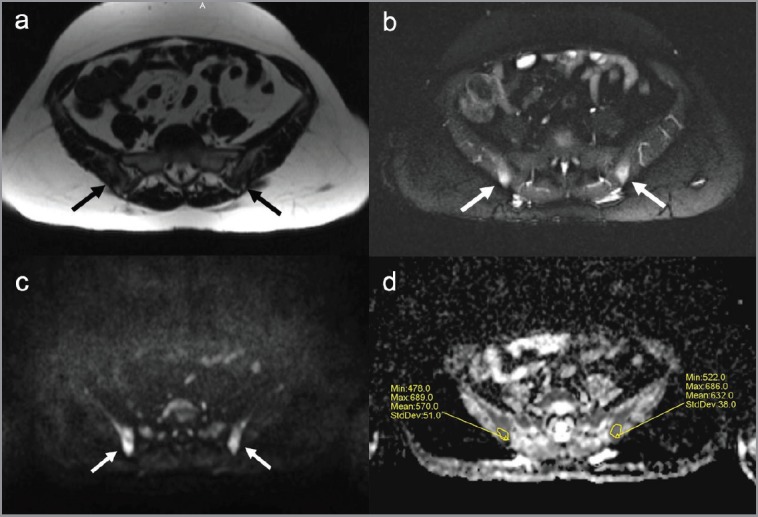
Pelvic bone lesions on diffusion whole-body MRI. Diffusion whole-body MRI showed ill-defined abnormalities in the iliac bones (arrows). The lesions presented low signal on T1-weighted images (a) high signal on STIR images (b) and on diffusion-weighted images with b-value of 900 s/mm^2^ (c), impeded diffusion of water molecules in ADC map with ADC values ranging from 478 to 686 µm^2^/s (d).

**Figure 6.**
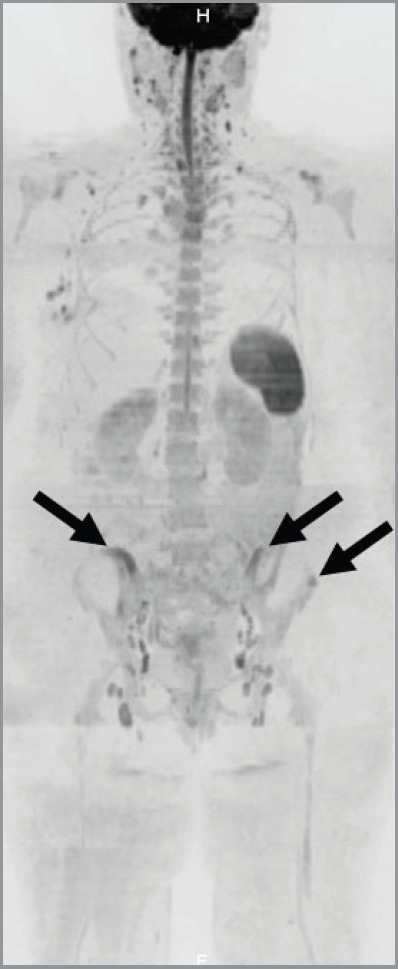
Maximum-intensity projection of diffusion-weighted images. Inverted maximum-intensity projection reconstruction of diffusion-weighted images (b-values, 50–900 s/mm^2^) showed high signal lesions in the pelvis (arrows).

**Figure 7.**
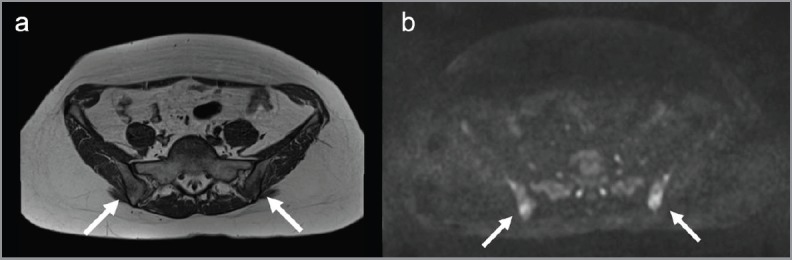
Follow-up MRI. (a). MRI of the pelvis showed the lesions (arrows) not significantly increased in dimensions on T1-weighted images and (b). diffusion-weighted images with b-value of 900 mm/s^2^.

Diffusion whole-body MRI showed ill-defined lesions in signal intensity in the iliac bones and a round lesion in the left iliac crest, with low signal intensity in T1-weighted images (Figure 5), and high signal intensity in T2-weighted (Figure 5) and diffusion-weighted images (Figure 5 and 6). The apparent diffuse coefficient (ADC) values were measured tracing polygonal region of interests (ROIs) within the inner contour of each bone lesion in the ADC map. Lesions showed impeded diffusion of water molecules with ADC values ranging from 478 to 686 µm^2^/s (Figure 5). The radiologist excluded the presence of metastatic disease and reported such bone lesions as benign, interpreting them as inflammatory abnormalities related to sarcoidosis. At four months, an MRI of the pelvis including diffusion-weighted imaging showed stable size and imaging characteristics of the lesions (Figure 7), thus supporting the diagnosis of inflammatory bone abnormalities. 

## Discussion

Multifocal skeletal sarcoidosis may present as a false positive for bone metastases on ^18^F-FDG PET/CT, as described in case reports [5, 6, 7], since granulomatous bone marrow infiltration may have an uptake of ^18^F-FDG which mimics that of metastatic disease [8, 9].

When false positive findings on ^18^F-FDG PET/CT cannot be totally excluded, biopsy or MRI may represent the second choice to achieve diagnosis. In our case, no lesion available for biopsy was visible at preliminary CT scan. Since it is well known that metastases in the bone marrow may not always be revealed on CT, thus making CT-guided biopsy difficult, our patient was referred to further investigation with MRI. Since conventional MRI may not be accurate in distinguishing between sarcoidosis and metastatic bone lesions we performed diffusion whole-body MRI [4]. Our protocol for diffusion whole-body MRI included both conventional MR (T1-weighted, T2-weighted STIR) and diffusion-weighted imaging (b-values: 50–900 s/mm^2^). The latter is able to evaluate microscopic tissue water motions average at the mm scale of MR images. The ADC value reflects the degree of freedom of water movement at the cellular level, which is determined by architectural tissue properties such as cellular density, cellular arrangements, vascularity, extracellular space tissue viscosity, and nuclear/ cytoplasmic ratio [10]. Water movement is impeded in many tumours because of their high cellular density and T2 relaxation times, resulting in high signal intensity on diffusion-weighted images and low ADC values [11].

On conventional imaging the pelvic bone lesions appeared with a signal pattern not specific for sarcoidosis bone lesions or metastatic disease (low signal on T1-weighted images and high signal on STIR images) [2]. On diffusion-weighted imaging the pelvic bone lesions showed high signal, which is often seen in bone metastases, but the ADC (<700 µm^2^/s) was too low to be suspicious for metastases from breast cancer. The largest series study published with a technique comparable to ours showed that bone metastases from breast cancer usually have higher mean ADC values (942 ± 154 µm^2^/s) with a cut-off value of 774 µm^2^/s, which enables to differentiate normal bone marrow from malignant marrow [12].

In relation to the clinical history (documented active sarcoidosis) and imaging appearance (negative CT, high-signal lesions on diffusion-weighted imaging with ADC values below the threshold for malignancy) we considered our findings as benign, more compatible with inflammatory changes related to sarcoidosis, and we put the patient under imaging follow-up. The follow-up MRI at four months interval which included diffusion-weighted imaging showed stable size and imaging features. Thus our diagnosis of benign lesions was further supported at follow-up.

## Conclusion

In our case, diffusion whole-body MRI proved to be a useful non-invasive and radiation-free technique for bone assessment in this cancer patient, which has helped in overcoming the limitation of ^18^F-FDG PET/CT in discriminating inflammatory bone marrow involvement from metastatic disease. However, large-scale studies still need to be done to investigate whether diffusion-weighted imaging is reliable in differentiating inflammatory bone marrow involvement, such as in sarcoidosis from metastatic disease.

## Conflicts of interest

The authors declare that they have no conflict of interest.

## Authors’ contributions

GC: writing, literature review

FZ: writing, literature review

MC: patient’s management

GR: patient’s management

MB: writing

GP: patient’s management, writing, literature review
